# Diabetic Retinopathy May Covariate With Stroke in Diabetes Mellitus

**DOI:** 10.7759/cureus.28227

**Published:** 2022-08-21

**Authors:** Meetali Kalani, Pranaykumar Shinde

**Affiliations:** 1 Ophthalmology, Datta Meghe Institute of Medical Sciences, Wardha, IND; 2 Ophthalmology, Jawaharlal Nehru Medical College, Datta Meghe Institute of Medical Sciences (Deemed to be University), Wardha, IND

**Keywords:** macular oedema, stroke, cataract surgery, femtosecond laser technology, neurodegeneration, hyperglycemia, ocular morbidity, endophthalmitis, postoperative pseudophakic cystoid macular oedema, diabetes mellitus

## Abstract

Diabetes mellitus is a chronic metabolic disorder with increasing prevalence per hour. Cataracts are one of the most common eye complications, and they affect all structures of the eye. The incidence of cataracts is increasing in patients with diabetes by several mechanisms. With the advancement of technology, cataract surgery is now a necessary procedure for diabetic patients. High-risk complications, like diabetic macular oedema, diabetic retinopathy (DR), phakic, postoperative cyst, and postoperative macular oedema, and macular oedema and endophthalmitis following surgery for a pseudocyst, could result in blindness. The importance of preoperative, intraoperative, and postoperative factors cannot be overestimated in managing complications and improving visual outcomes. DR can be a severe problem if it worsens and causes non-proliferative or proliferative DR or if fluid accumulation in the eye is diagnosed as macular oedema. A woman progressing to sight-threatening DR during childbearing age experiences distress and often requires ocular treatment. Diabetes that has been present for a more extended period, as well as more significant hyperglycaemia, hypertension, cardiovascular diseases, and elevated blood pressure, substantially predict the development of DR. Oxidative stress can be caused by hyperglycaemia, irregular metabolic processes, and people with DR developing neurodegeneration. Therefore, controlling postprandial hyperglycaemia is crucial for preventing DR. Femtosecond laser technology, multifocal intraocular lenses, and other surgical innovations are popularly referred to as surgical management; it will be engaged in the coming era to determine whether there will be a continued reduction in the complication of cataract surgery. This article aims to review the correlation of DR with stroke and its screening and to outline the critical management strategies.

## Introduction and background

According to International Diabetes Federation, more than 285 million people are affected worldwide by diabetes mellitus (DM), and this number is expected to rise almost twofold to 439 million by 2030. There is a two to three times higher risk of developing diabetic retinopathy (DR) in diabetic patients. There is evidence that DR can lead to neurodegeneration, and it is suggested that this may coincide with and cause retinal vasculopathy. There is a reduction in the number of nerve cells in the retina and in the layer of cells that helps to form the retina's ganglion and Muller cells. Cataracts are considered the most significant cause of visual impairment and lead to changes in the optics of the diabetic eye. In some clinical studies, it is evaluated that up to 2% of cataract surgeries are performed on patients with diabetes, and it is also reported that cataract surgery leads to a rapid progression of DR, provokes bleeding into the vitreous body, causes iris neovascularization, and eventually leads to reduction or loss of vision [1]. There is still much debate about whether or not diabetic patients improve after cataract surgery. Some studies show that the surgery is successful, while others show that the patients experience complications [2]. A cataract is a cloudy or opaque area in the eye's lens leading to changes that can impair vision [3]. It is a complex condition influenced by age, genetic predisposition, smoking-related illnesses, DM, drug use, and environmental exposure. The quality of life suffers due to a significant decrease in visual acuity. Therefore, surgery is performed to remove the opacified lens [4]. DR is a major complication after cataract surgery, and its prevalence rate is also rising in the elderly. Additionally, DR is progressively contributing to visual loss in the older population. Its high incidence is linked to diabetes duration and inferior glycaemic control. Its high incidence is associated with long-term diabetes, particularly inadequate glycaemic management. DR is analogous to an increased risk of vision loss in older people. Diabetes is higher in people who have been diagnosed with the condition for a longer time and have poor glycaemic control. Diabetic microvascular complications often occur together, such as diabetic nephropathy. There is a problem with the endothelial cells that line the blood vessels in people with diabetes, which can lead to kidney problems and blood clots. There is a decrease in macular blood flow in diabetic retinas, likely due to altered blood flow regulation [5]. The most frequent cause of visual loss in people with DR is diabetic macular oedema. An increased risk of diabetic macular oedema is linked to an increase in the prevalence of DR. A rise in the incidence of diabetic macular oedema is linked to an increase in the prevalence of DR [6]. The most important factors leading to the development of diabetes are the duration of the disease, high blood sugar levels, high body weight, puberty and pregnancy, and cataract surgery. Some studies have shown the relationship between blood glucose level and onset and progression of DR. Also, correspondence between high blood pressure and DR is well established [7]. Previous studies have related stroke and polygenic disorders and have had loads of attention in recent years. The incidence of stroke is two to three times higher in individuals with diabetes compared to non-diabetic individuals. Diabetes retinopathy will be enclosed in a routine examination for chronic complications of diabetes. There is evidence that microvascular illness contributes significantly to stroke, and as DR is considered a diabetic microvascular consequence, it is the only ocular component associated with stroke [8]. During the early stage of the disease, patients are usually asymptomatic; however, there is a progression over time with the development of microaneurysm and microvascular haemorrhages in brain anomalies. If therapy is ineffective, there is evidence of bruising, blurred vision, impaired colour vision, and possibly vision loss. Certain people have a severe type of DR, where there is the creation of retinal blood vessels that are very absorbent. The first clinically recognizable feature in the development of DR is the presence of tiny red dots scattered in the retina posteriorly. It may be surrounded by a thin ring of yellowish lipid or hard exudates, known as microaneurysms. They resemble balloon-like capillary wall protrusions and are known to activate inflammatory cells, which also harm the endothelial cell lining. It is said that late-degree microaneurysms are sclerotic and that they typically exist without endothelium lining, and they are associated with significant capillary degeneration in certain locations [9].

## Review

DR

DR is a term used to describe the microvascular deformity that is observed in the fundus of diabetic patients [10]. It is classified according to the main criteria for microvascular lesions. It is divided into two broad categories: proliferative DR (PDR) and non-proliferative DR (NPDR). The feature of preretinal neovascularization characterizes PDR. NPDR is represented on the basis of clinical findings, including microaneurysms, retinal haemorrhages, venous changes in venous calibre and abnormalities in intraretinal microvasculature. In PDR, progressive capillary nonperfusion and ischemia that results from it are followed by hypoxia, which in turn stimulates the growth of abnormal new blood vessels in the retina that protrude in front of the retinal pigment epithelium retinal void [11]. The dysfunction of the blood-retinal barrier (BRB) is common in DR, which could cause leakage of blood components and circulating proteins to nerve cells. Due to the destruction of internal BRB, the retina causes diabetic macular oedema. This causes an abnormal thickening of the retina and cystic oedema of the macula [9][11]. Diabetes can result in potentially sight-threatening DR, such as sight-threatening diabetic macular oedema or severe onset of NPDR. Numerous studies have demonstrated that pregnancy carries a unique risk for the development of DR. The effect of sight-threatening DR (STDR) leads to ocular morbidity during pregnancy and causes distress to the reproductive mother. Follow-up is essential for people with a long duration of DM, poor initial glycaemic control, and severe DR, as these can lead to the progression of STDR [12]. The Early Treatment DR Study has classified the stages of DR based on vascular alterations, including dot/blot haemorrhages, hard/soft discharge, intraarterial microvascular anomalies, and angiogenesis. The development and progression of diabetic macular oedema are related to an increase in vascular permeability [13]. According to particular research, persons with diabetes have a much higher risk of stroke than those who do not have the disease. Additionally, several analysts concluded that having DR increases the risk of stroke in people living with diabetes [8]. Consequently, routine DR screening should be carried out. Optical coherence tomography (OCT) helps assess and control the risk of stroke; additional ophthalmological procedures include angiography and scanning laser ophthalmoscopy examination [8]. The stages of DR and its clinical findings are mentioned in Table 1.

**Table 1:** The Stages of Diabetic Retinopathy and Its Retinal Finding.

STAGES OF DIABETIC RETINOPATHY	RETINAL FINDINGS
Mild non-proliferative diabetic retinopathy	Microaneurysms.
Moderate non-proliferative diabetic retinopathy	At least one of the following is present: retinal haemorrhages, cotton wool spots, hard exudates.
Severe non-proliferative diabetic retinopathy	Any of the following but no signs of proliferative diabetic retinopathy: intraretinal haemorrhages, venous beading, intravascular microvascular abnormality.
Proliferative diabetic retinopathy	Either of the following is found: preretinal haemorrhage, neovascularization.

Clinical manifestation

The earliest and most serious manifestation is punctate microaneurysms, which are localized saccular ridges on the walls of capillaries appearing as small red dots with sharp edges. Another clinical feature is retinal haemorrhages, which are present throughout the fundus. Retinal haemorrhages can take on a flame-like look instead of a punctate or patchy appearance when they occur in the nerve fibre layer rather than the middle layer of the retina [10]. PDR is characterized by angiogenesis on the retina, optic disc, or posterior vitreous surface. Diabetes-related retinopathy's onset increases perinatal follow-up visits during pregnancy [11]. The likelihood of developing diabetes is correlated with the severity of retinopathy during the first year after surgery and macular oedema. The indications of diabetes macular oedema are characterized by retinal thickening brought on by blood vessel leakage. This can occur at any point during diabetic retinal disease [11]. The diabetic cornea has a significant risk of developing epithelium abnormalities, reduced sensitivity, aberrant wound healing, epithelial basement membrane dysfunction, and increased susceptibility to injury and ulceration because of this impairment. They also demonstrate impaired growth production, alteration in angiogenic response, and collagen accumulation in the diabetic cornea, which eventually leads to inappropriate corneal wound healing [14]. Since DR is associated with both microvascular and macrovascular issues, it is thought that cardiovascular autonomic neuropathy contributes significantly to diabetic sequelae and autonomic neuropathy. Diabetic patients present with endothelium dysfunction, which can result in cardiovascular dysfunction, primarily affecting coronary, peripheral, and carotid arteries [15]. Ischemic and haemorrhagic strokes can occur in DM, but hemorrhagic stroke is less common. Several studies have examined the circulation in the circle of Willis arteries and posterior and anterior circulation. Patency in the circle of Willis frequently appears in DM along with posterior circular brain infarction and brain stem infarction. In those with DM, intracranial stenosis is a substantial risk factor for ischemic stroke. Since a greater risk of dementia is associated with middle cerebral artery stenosis, Type 2 diabetic individuals are more susceptible to vascular death. Other exciting studies have demonstrated that among individuals with Type 2 DM, asymptomatic middle age is connected with albuminuria and hypertension [16]. All clinical forms of diabetes, obstructive arterial disease, severe cerebrovascular illness, considerable dysfunction, and a shorter life expectancy have been linked to them. In a large meta-analysis of 102 studies, DM was associated with a risk for ischemic stroke that was 2.27-fold higher than in those who do not have diabetes [17].

Pathophysiology of stroke

A highly complex interplay exists between the brain and cerebrovascular events to meet the brain's metabolic demands and enable it to function properly. DM is responsible for the proper functioning. It is also accountable for the pathologically caused destruction of cerebral artery structure, angiogenesis and vascular regression, as well as changes in the cerebrovascular area functions leading to compromised myogenic reactivity and endothelial damage malfunction [18]. The remodelling and structure of the cerebrovascular system are both affected by metabolic illnesses; these alterations may raise the risk and delay the healing and functional consequences of stroke. The superficial brain is perfused by meningeal arteries, including coronary arteries and collateral circulation, and furnishes the brain's surface. Type 2 DM remarkably increases the intra-tree-arteriole-to-arteriole connection between the anterior trunk and mediastinum protection of the carotid artery and blood-brain barrier by lowering blood pressure splicing proteins. It is also associated with a rapid decline in neurovascular function coupling. Neurovascular connection is necessary for the cerebrovascular system to maintain the homeostasis of the brain's environment. This connection is compromised, resulting in the energy substrates required for neural activation not being delivered as effectively [18]. Several mechanisms have been associated with stroke in DM, including increased early arterial stiffness, vascular endothelial dysfunction, systemic inflammation, and basal capillary membrane thickening. Hyperglycaemia and insulin resistance are the major factors responsible for the development of atherosclerosis. Free fatty acids and inflammatory cytokines are end products from adipose tissues, which leads to reduced lipid metabolism, which causes increased production of reactive oxygen species and ultimately leads to an increase in systematic inflammation. There is a decrease in the endothelial synthesis of nitric acid and production of nitric oxide (NO); as NO plays an essential role in maintaining the function of endothelial cells, its reduction causes endothelial dysfunction. Recurrent hypoglycaemia may also be considered a risk factor for stroke [16]. Neurodegeneration is characterized by apoptotic loss of retinal neurons. Inflammation is a systematic response against different agents and involves a cascade mechanism. The reciprocal balance between pro-inflammatory and anti-inflammatory stimuli facilitates the healing process. Here, we are paying attention to DM with prolonged homeostasis inflammation leading to irreversible tissue damage [19]. DR and stroke have a vascular relationship. Retinoscopy is a macrovascular dysfunction due to endothelial dysfunction, which results in leakage from the artery wall. It causes lipid accumulation, thereby leading to an episode of atherosclerosis [20]. Diabetic patients with DR have more symptoms and increased cardiovascular dysfunction, including a 70% risk of thromboembolic stroke events [21]. Myocardial death has an essential effect on endothelial dysfunction and stroke pathophysiology. There is reticular endothelial stress which promotes apoptosis and cellular damage. Apoptosis, autophagy, and cellular necrosis are caused by ER stress, oxidative stress, inadequate calcium handling, and autophagy [15]. Strokes in patients with DM are usually due to atherosclerosis, hypertension, and arterial fibrillation. Three main mechanisms responsible for ischemic strokes are hypercoagulable state, vasculitis, and cardiomyopathy [22]. In ischemic stroke, estrogen is highly neuroprotective, inhibiting pathological stroke risk factors through antiatherogenic effects in vascularization and lipogenesis. When there is ischemia, estrogen reduces the risk of stroke by preventing coronary artery dilatation and protecting the brain and glial cells from damage [23]. Hyperglycaemia is often a clinical presentation in patients with acute stroke. High blood glucose levels show previously undiagnosed prediabetes or even diabetes. A stroke can cause a critical stress response that stimulates the hypothalamus-pituitary-adrenal axis, ultimately leading to the release of catecholamines, glucagon, and cortisol. Therefore, some theories suggest that post-stroke hyperglycaemia is caused by an inflammatory response [24].

Cerebral cardiac syndrome

Cerebral cardiac syndrome (CCS) is a condition known as vasovagal syncope that involves the connection of the heart and brain. Even without initial cardiac problems, this can be significant following brain damage. However, it is more likely to happen in the history of cardiovascular illnesses and brain problems, including obesity, diabetes, and hypertension. Significant brain damage is an ischemic stroke. Ischemic stroke patients with cardiac dysfunction have a high mortality rate, and hospital stays longer than patients without cardiac complications. Clinical characteristics of CCS include arrhythmia, myocardial damage, and heart failure. The degree, duration, and clinical features should be noted to advertise CCS. The sensitivity of both eyes is increased by DR. The heart and brain are at an advanced stage. Still, it is also known to induce systemic oxidative and hyperosmolar stress stimulation due to inflammation and more heart inflammatory injury in diabetes stroke [25].

Screening and diagnosis of DR

Detection of DR is performed in two steps: screening and diagnosis [26]. To prevent irreversible vision loss, screening for DR is essential to find the root causes that need prompt, thorough ocular examination and treatment. Individual screening intervals have been shortened during the previous few years. With careful cost-efficiency, various risk variables are offered for utilization ratios. Resources for countrywide screening programs are however limited. Innovative techniques such as wearable technology, remote ophthalmology for remote grading, computer science for autonomic function, and confocal medical scanning with ultrawide (UW) field imaging make up dynamic screening methods to better identify and grade DR at a lower cost. In addition, growing research points to the possibility that retinal imaging could indicate a person’s risk of psychological impairment in traits that could make diabetes more noticeable when screening for retinopathy while it does not yet pose a threat to visual disease [27]. The development of OCT as a useful DR diagnostic tool has enabled instantaneous sophisticated two- and three-dimensional screening, and accurate mapping of histopathological changes with opalescent retinal structures. It is very fast and provides quantitative assessment with focused scans. The use of OCT in diagnosing and treating diabetic eye disease has benefited our understanding of how diabetes affects the retina's fundamental structure. Seventy-three to seventy-five percent of OCT have substantially advanced the cause of DR patients by allowing direct analysis and quantitative retinal thickness monitoring, where the changes in thickness will greatly influence therapeutic decisions [28]. Inflammatory processes have beneficial and detrimental impacts on the progression of ischemic brain damage [29]. Fluorescein angiography and colour fundus imaging with UW-field OCT angiography and imaging are two methods that can be utilized for DR imaging. Retinal perfusion and vascular leakage are evaluated by angiography. Also, physicians use UW imaging of the macula and posterior pole to understand the severity of retinopathy across the retina [30].

Management of DR

Ischemic stroke risk is correlated with having DM. Medical interviews and physical, neurological, and tomography examinations are performed to diagnose stroke. CT, MRI, magnetic resonance angiography (MRA), CT angiography, and artery imaging are used to classify the form of ischemia stroke. Immediate treatment or endovascular medical air improves the prognosis. Antiplatelet therapy is suggested for functions of risk reduction of repeated stroke. However, glucose management is uncertain [31]. Nephropathy and high blood pressure can also aggravate retinopathy and require stabilizing treatment. Careful collaboration with anesthesiologists to treat perioperative hypertension pressure can lower troubles with intraretinal bleeding. Anti-vascular endothelial growth factor (anti-VEGF) drugs and corticosteroids can also stabilize DR [32]. Recent tendencies in computer-aided device techniques, which might be described within the scope of artificial intelligence, are seeing more use in cutting-edge ophthalmology as they can keep a record of time, price and human assets for regular DR screening and contain decreased diagnostic blunders factors [26]. The action to control cardiovascular risk in diabetes (ACCORD) experiment was created to examine the impact of extended glycaemic control on cardiovascular health and microvascular incidents in Type 2 diabetic individuals [33]. The first automated DR screening program was approved by the FDA in April 2018; thanks to data analytics, artificial intelligence and, a deep learning algorithm, it has achieved a 96.8% sensitivity and 87% specificity for recognizing referable DR [34]. There is currently no known treatment for DR. The control of the underlying causes and risk factors tends to be the only treatment that makes sense. These include maintaining optimal blood pressure management and blood sugar regulation. Additionally, it's essential to highlight other risk factors including nephropathy and anaemia. Laser photocoagulation is one surgical technique that has proven a good prospect in the treatment of DR. The primary goal of this approach is to repair the capillary damage that is present in DR patients. This approach has shown a lot of promise because it not only slows the disease's course but also greatly reduces the amount of visual loss experienced by retinopathic patients [35].

## Conclusions

DR is a bothersome complication of DM; the pathophysiology, causation, development, and treatment options are poorly known and explored. To unravel the DR riddles, more investigation is required. Treating this illness is quite difficult due to the lack of research and therapy alternatives. Therefore, additional research is required to further our understanding of this condition and to investigate the best treatment options for this burdensome diabetic complication. In order to preserve good vision and prevent laser photocoagulation, it might be very helpful to stop the start of DR or slow its progression. Normalizing blood sugar levels, strict blood pressure control, lowering low-density lipoprotein (LDL) cholesterol with statins, using fenofibrates, and blocking the renin-angiotensin system have all been demonstrated to be somewhat beneficial in preventing the development of DR. DR is an important complication of diabetes, and has high incidence and high mortality rate in diabetes patients. Stroke most commonly occurs following other cardiovascular events such as ischemia and hypertension. In conclusion, patients with diabetes who have DR status have a higher risk of stroke. However uncertain in Type 1 DM patients, this association was significant in Type 2 DM patients. Therefore, utilizing OCT, OCT angiography, scanning laser ophthalmoscopy, and other ocular tests, screening for DR should be considered a routine step in the assessment and treatment of stroke risk.
